# Chemical Conditioning as an Approach to Ischemic Stroke Tolerance: Mitochondria as the Target

**DOI:** 10.3390/ijms17030351

**Published:** 2016-03-08

**Authors:** Zhen Jin, Jinzi Wu, Liang-Jun Yan

**Affiliations:** Department of Pharmaceutical Sciences, UNT System College of Pharmacy, University of North Texas Health Science Center, Fort Worth, TX 76107, USA; zhen.jin@unthsc.edu (Z.J.); jinzi.wu@unthsc.edu (J.W.)

**Keywords:** chemical conditioning, ischemic conditioning, hypoxic conditioning, mitochondria, neuroprotection, stroke injury

## Abstract

It is well established that the brain can be prepared to resist or tolerate ischemic stroke injury, and mitochondrion is a major target for this tolerance. The preparation of ischemic stroke tolerance can be achieved by three major approaches: ischemic conditioning, hypoxic conditioning and chemical conditioning. In each conditioning approach, there are often two strategies that can be used to achieve the conditioning effects, namely preconditioning (Pre-C) and postconditioning (Post-C). In this review, we focus on chemical conditioning of mitochondrial proteins as targets for neuroprotection against ischemic stroke injury. Mitochondrial targets covered include complexes I, II, IV, the ATP-sensitive potassium channel (mitoKATP), adenine dinucleotide translocase (ANT) and the mitochondrial permeability transition pore (mPTP). While numerous mitochondrial proteins have not been evaluated in the context of chemical conditioning and ischemic stroke tolerance, the paradigms and approaches reviewed in this article should provide general guidelines on testing those mitochondrial components that have not been investigated. A deep understanding of mitochondria as the target of chemical conditioning for ischemic stroke tolerance should provide valuable insights into strategies for fighting ischemic stroke, a leading cause of death in the world.

## 1. Introduction

The brain is a vulnerable target of ischemic stroke injury, but can also be made to resist or tolerate such an injury [1,2,3,4,5]. Brain ischemic tolerance can be achieved by a variety of approaches [6,7,8], of which the most extensively-studied ones are conditioning that encompasses preconditioning (Pre-C) and postconditioning (Post-C) [9,10,11,12]. Under the concept of conditioning, further categories can be classified depending on the design of conditioning induction. When conditioning is achieved by short cycles of ischemic reperfusion, the strategy is known as ischemic conditioning [13,14]. When conditioning is achieved by one cycle or many short cycles of hypoxic exposure, the strategy is known as hypoxic conditioning [15]. If the conditioning is achieved by chemicals or pharmacological drugs that usually target proteins, such an approach is often called chemical hypoxia or chemical conditioning [16]. It should be noted that both hypoxic conditioning and chemical conditioning are cross-tolerance approaches [17], as the induction of ischemic tolerance is not achieved via ischemia. Importantly, regardless of which category of conditioning, both Pre-C and Post-C strategies under a given conditioning approach involve triggering the endogenous neuroprotective responses during the conditioning phases that can be in full-fledged action upon subsequent severe ischemic injury [11,18,19,20]. Mitochondria are known to be involved in ischemic stroke injury and chemical-induced ischemic tolerance [21,22,23,24]. Therefore, in this review, we first discuss the role of mitochondria in ischemic injury, ischemic conditioning and hypoxic conditioning and then focus on mitochondrial protein targets that have been widely studied in the context of chemical conditioning and neuroprotection in ischemic stroke injury.

## 2. Mitochondria and Ischemic-Reperfusion Injury

Mitochondria are known to play a major role in ischemic/reperfusion injury [25,26,27]. During ischemia, ATP production by mitochondria is dramatically deceased due to the lack of nutrients and oxygen [28]. This decrease in ATP levels leads to impairment in ATP-dependent Ca^2+^ channels, resulting in accumulation or overload of cellular and mitochondrial Ca^2+^ [28]. In the meantime, lack of blood flow also causes accumulation of lactate, as cells are forced to undergo anaerobic respiration [28]. Consequently, cellular pH decreases, which induces the closure of the mitochondrial membrane permeability transition pore [28]. Upon reperfusion, a sudden resupply of oxygen and nutrients re-energizes mitochondrial aerobic respiration, resulting in a further load of mitochondrial Ca^2+^, mitochondrial permeability transition pore (mPTP) opening and a burst in reactive oxygen species (ROS) production [29,30,31]. Cytochrome c release due to mPTP opening would set off the cellular apoptotic process [32,33], and ROS generation would cause widespread oxidative damage to macromolecules, such as DNA, lipids and proteins [34,35,36,37,38,39]. The occurrence of these events converging on mitochondria would eventually lead to cell death and tissue infarction [40]. Therefore, mitochondria have been investigated as a major target for neuroprotection in ischemic brain injury [20,41,42,43].

## 3. Ischemic Conditioning: Ischemic Pre-C and Post-C

**Figure 1:**
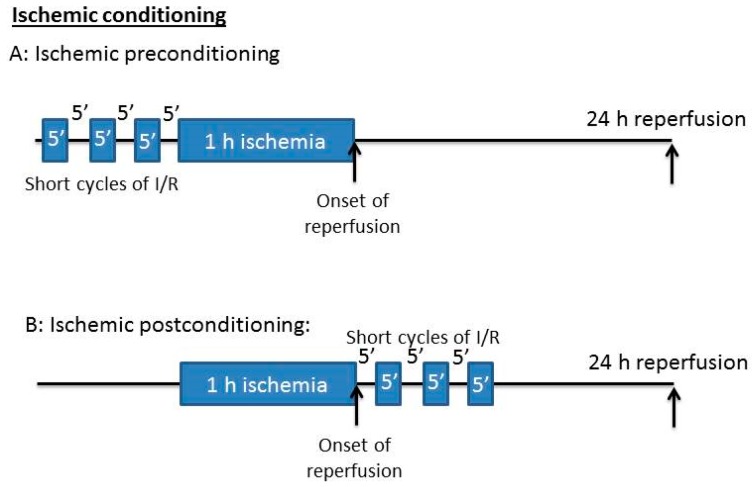
General scheme of ischemic preconditioning and ischemic postconditioning. The short cycles of ischemia reperfusion can be used either preconditioning induction (**A**) or postconditioning induction (**B**). As there are no standard procedures for ischemic conditioning, the number of cycles varies widely from investigator to investigator.

In ischemic Pre-C, the brain is subjected to short episodes of ischemic reperfusion followed by a severe ischemic stroke attack [44,45,46] (Figure 1A). In ischemic Post-C, the reperfusion process following a period of ischemia is interrupted by several short cycles of ischemia [11,12,47] (Figure 1B). This approach, though developed much later than that of ischemic Pre-C, has gained great attention and momentum in the field of stroke research. The reason is that stroke is really an unpredictable mishap, so Post-C is more clinically relevant than Pre-C. Nonetheless, Pre-C still remains an intensively-studied area because it can be manipulated to understand the endogenous neuroprotective mechanisms [48,49,50]. It should be noted that the cycle number for the short episodes of ischemic reperfusion conducted before or after lethal ischemic stroke injury can vary considerably depending on the purpose of a study.

## 4. Hypoxic Conditioning: Hypoxic Pre-C and Post-C

**Figure 2:**
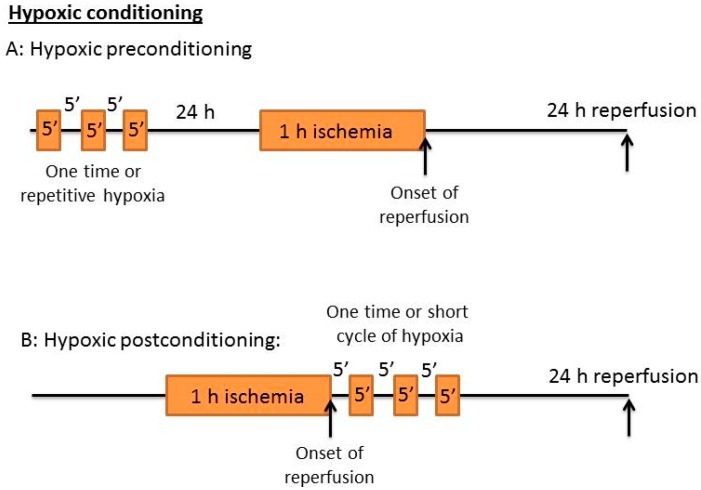
Representative scheme of hypoxic preconditioning and postconditioning. Similar to that of ischemic conditioning, the short cycles of hypoxia exposure can be applied either before (**A**) or after (**B**) lethal stroke. There are also no standard procedures for hypoxic conditioning, so the protocol could change significantly from laboratory to laboratory.

Similar to ischemic Pre-C and Post-C, hypoxic Pre-C and Post-C have also been widely used as neuroprotective approaches [51,52]. Shown in Figure 2 are the general approaches of hypoxic exposure conducted either before or after lethal ischemic stroke. As there are no standard procedures for hypoxic exposure, the schemes in Figure 2 only show representative procedures that can vary from investigator to investigator. Under many conditions, repetitive hypoxic exposure, whether Pre-C or Post-C, is performed [53].

## 5. Chemical Conditioning: Chemical Pre-C and Post-C

**Figure 3:**
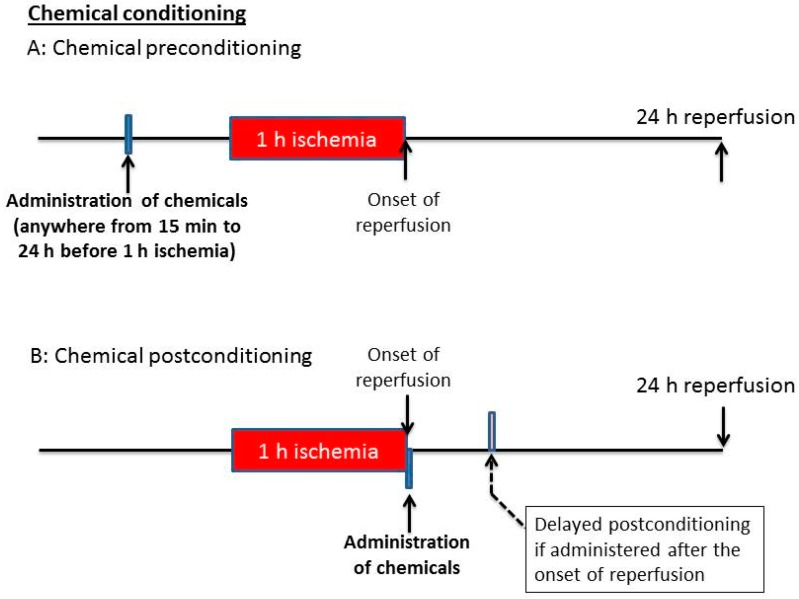
Representative scheme of chemical conditioning. The administration of the chemical agent or pharmacological drugs can be given either before (**A**) or after (**B**) lethal ischemic stroke. If the chemical is given during the reperfusion process instead of at the onset of reperfusion, delayed postconditioning effects would be induced.

It has long been known that chemicals or pharmacological agents can be used to make the brain resistant to ischemic stroke injury [54,55,56]. The process of chemically-induced ischemic stroke tolerance is a cross tolerance [57], as the induction of conditioning is not achieved by ischemia, but by chemicals or pharmacological agents. Chemically-induced ischemic tolerance is also often called chemical hypoxia [58,59]. This is because the use of chemicals at a sublethal dosage always impacts cellular mitochondrial respiration, whereby cells work under hypoxic conditions. The conditioning process can stress cells, but does not impair cellular function or lead to cell death, thereby preparing cells to prevent from further lethal ischemic stroke injury. General approaches of chemical conditioning, including both Pre-C and Post-C, are shown in Figure 3. In Pre-C settings, a chemical is administered before ischemic stroke so that a preventive purpose could be served. In Post-C settings, a chemical is administered at the onset of reperfusion, whereby the reperfusion process is disrupted or interfered with, so that less tissue damage could be achieved. If the induction agent is administered after reperfusion has started, say a few hours after the onset of reperfusion, such an approach would be known as delayed chemical conditioning [60]. We think chemical conditioning is more feasible than ischemic or hypoxic conditioning because a given chemical can be readily administered via injection or inhalation without needing any other equipment or instruments.

## 6. Components of Mitochondrial Metabolic Pathways as Targets of Chemical Conditioning

**Figure 4:**
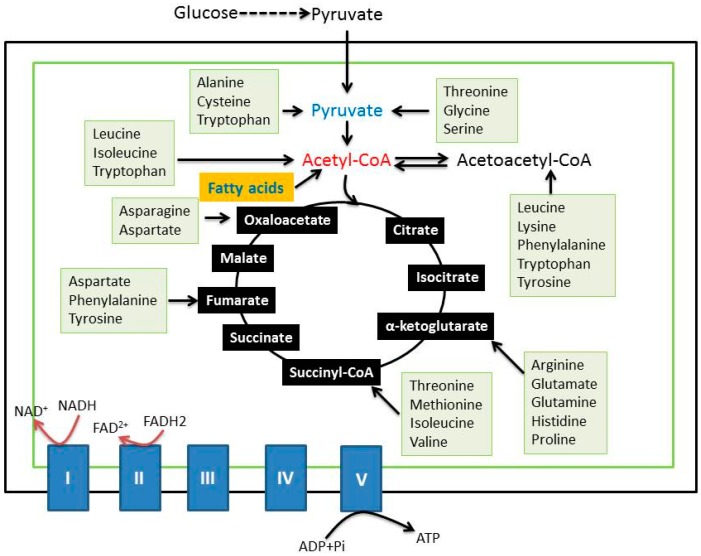
Overview of mitochondrial metabolic pathways that could be potential targets for chemical conditioning for neuroprotection against ischemic stroke injury. These pathways mainly include glucose metabolism, fatty acid oxidation, amino acid breakdown, the Krebs cycle and the electron transport chain, as well as oxidative phosphorylation. Numerous enzymes in these pathways have not been explored in terms of chemical conditioning and stroke protection, providing great opportunities for future studies of chemical conditioning and stroke protection.

**Figure 5:**
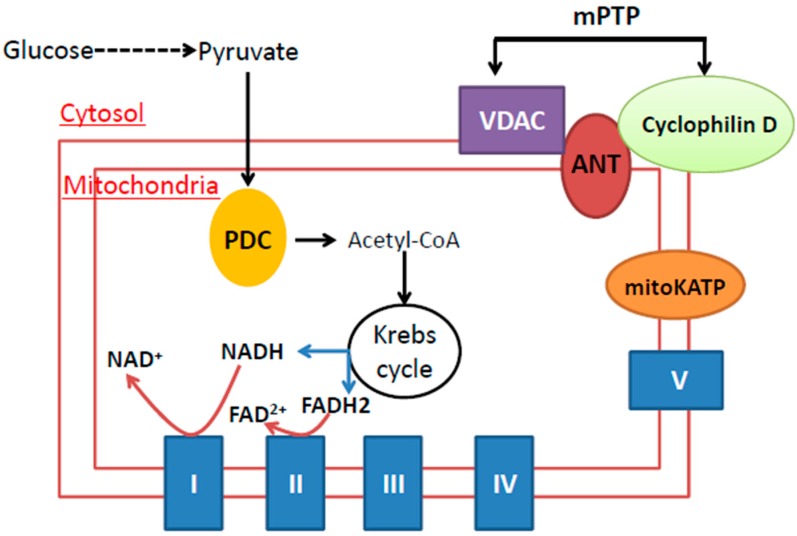
Mitochondrial targets covered in this review. These targets include complexes I, II and IV, the mitoKATP channel, adenine dinucleotide translocase (ANT) and the mitochondrial permeability transition pore comprised of ANT, the voltage-dependent anion channel (VDAC) and cyclophilin D. Please see the text for details.

In ischemic conditioning and hypoxic conditioning, there is often no specific target that could be defined. In contrast, when chemical conditioning is conducted, the target is often known because the chemical or compound is used to either inhibit or activate the function of a protein. When it comes to mitochondria as the target for chemical conditioning, literally, any proteins in mitochondria can serve as chemical conditioning targets as long as an inhibitor or activator of such a target exists or can be artificially synthesized. Obviously, the components of the mitochondrial metabolic pathways are ideal targets due to their involvement in oxygen consumption and nutrient electron extraction, as well as electron storage in nicotinamide adenine dinucleotide (NADH) and flavin adenine dinucleotide (FADH_2_) [61,62]. As shown in Figure 4, the mitochondrial metabolic pathways mainly involve the breakdown of pyruvate to form acetyl-CoA by pyruvate dehydrogenase, beta oxidation of fatty acids to also yield acetyl-CoA, amino acid residues’ catabolism to generate acetyl-CoA or the intermediates in the Krebs cycle and complete combustion of acetyl-CoA to carbon dioxide in the Krebs cycle. This is followed by electron transport from either NADH at complex I or FADH_2_ at complex II to oxygen via coenzyme Q and cytochrome c and, finally, ATP production at complex V [61,63]. All of the enzymes involved in these metabolic pathways could be potential targets of chemical hypoxia or chemical Pre-C or Post-C. Surprisingly, many of the enzymes involved in these metabolic pathways have not been investigated in the context of chemical conditioning and neuroprotection against ischemic stroke injury. However, the importance of these enzymes as possible chemical conditioning targets should not be discounted just because they have not been studied. The major mitochondrial components that have been studied as chemical conditioning targets and that are covered in this review are shown in Figure 5. These include complexes I, II, IV, the ATP-sensitive K^+^ channel (mitoKATP), adenine dinucleotide translocase (ANT) and the mitochondrial permeability transition pore (mPTP). In the following sections, we will summarize reported studies involving chemical conditioning of these mitochondrial components as stroke tolerance targets. As our purpose is by no means to exhaust the literature, we focus on the major mitochondrial targets that have been widely studied. Hence, we apologize to those whose work is not cited in this review. It should be noted that we have not found ample reports in the literature (mainly via PubMed searches) about chemical conditioning targeting complexes III and V for stroke tolerance.

### 6.1. Complex I Inhibition by Isoflurane

Isoflurane has been found to elicit both Pre-C and Post-C effects in many organs [64]. It is known to inhibit mitochondrial complex I [65,66,67,68,69], a major site for ROS generation [70,71,72,73]. In a Post-C study, Sosunov *et al.* [69] have found that isoflurane given upon reperfusion attenuated mitochondrial ROS production by inhibiting complex I function and the recovery of mitochondrial oxidative phosphorylation. The underlying mechanism involves a decreased hydrogen peroxide production, hence an attenuated oxidative stress in the brain and a decreased infarction volume. In contrast to the complex II and ANT preconditioning studies discussed below, this study indicates no positive roles of ROS, as ROS release from complex I was shown to be the main culprit of oxidative damage in the brain. As the study used neonatal mice as its animal model, whether the age of the mice could contribute to the deleterious role of ROS in neonatal mice remains unknown. Using adult rabbits, however, Ludwig *et al*. [74] have reported that isoflurane-mediated preconditioning in the brain involves ROS production at the site of complex III. It should be noted that isoflurane may also indirectly target other mitochondrial proteins or pathways [75]. For example, isoflurane Post-C may involve its inhibition of the mitochondrial permeability transition pore in neonatal rat brain [76]. It should also be noted that rotenone [77,78], a widely-used complex I inhibitor, has not been tested in the context of chemical conditioning and brain stroke tolerance, which is probably due to its toxicity [79,80]. The same is also true for antimycin A, a well-known complex III inhibitor [81,82].

### 6.2. Inhibition of Succinate Dehydrogenase (Complex II) by 3-Nitropropionate

3-Nitropropionate (3-NPA) is a well-known inhibitor of complex II that is the only complex sitting in both the Krebs cycle and the electron transport chain [83]. 3-NPA is an irreversible complex II inhibitor [84] and has been well studied in Pre-C-induced neuroprotection, as well as cardioprotection [85,86]. A series of studies conducted by Dirnagl and his colleagues have demonstrated that 3-NPA inhibition of complex II inhibits mitochondrial oxidative phosphorylation and increases hypoxic tolerance, both in animal studies and in cell culture studies [54,56]. Importantly, the 3-NPA regimen used in their studies did not induce any detectable cell death or behavioral changes. The mechanisms of 3-NPA-induced ischemic tolerance are believed to involve ROS production, as the antioxidant dimethylthiourea, when administered before 3-NPA treatment, abolished 3-NPA’s neuroprotective effect [87], indicating that ROS are required for 3-NPA-induced preconditioning and neuroprotection.

### 6.3. Preconditioning of Cytochrome c Oxidase (Complex IV) by Cyanide

Cyanide is a well-known inhibitor of cytochrome c oxidase [88], so-called complex IV, that is the last component of the electron transport chain [89]. It has been reported that inhibition of complex IV by a sublethal dosage of sodium cyanide can prevent neurotoxicity by a lethal dosage of sodium cyanide [90]. This cyanide-induced preconditioning against cyanide-induced neurotoxicity is not cross tolerant and was manifested by the maintenance of mitochondrial membrane potential and increased levels of Bcl-2 and Bcl-XL, indicating preservation of mitochondrial function [90]. It should be noted that the authors did not explore whether cyanide-induced preconditioning has any protective effect against ischemic stroke injury that would be a cross tolerance study.

### 6.4. Inhibition of Adenine Nucleotide Translocase by Carbon Monoxide

Queiroga *et al.* [91] found that CO can elicit a protective response against astrocyte cell death induced by diamide, a thiol crosslinking agent that usually causes oxidative stress [92]. The authors found that CO works by directly enhancing ANT function via a mechanism of protein s-glutathionylation. As ANT is part of the mitochondrial permeability transition pore (mPTP) [93,94], the functional enhancement of ANT actually prevents ANT’s pore forming function, leading to no mitochondrial membrane swelling and no cytochrome c release. Additionally, CO preconditioning also involves ROS formation, as the use of β-carotene can abolish CO’s protective action. This study further confirms that ROS formation during preconditioning is essential for a preconditioning effect.

### 6.5. Inhibition of Mitochondrial Permeability Transition Pore by Carbon Dioxide

In an elegant study, Fan *et al*. [95] have found that carbon dioxide (CO_2_) also can elicit a Post-C neuroprotective effect. The authors found that stroked mice receiving varying dosages of CO_2_ at 5, 50 or 100 min after onset of reperfusion showed a pronounced neuroprotective effect after ischemic stroke injury. The protective effect of CO_2_ was found to be due to its induction of mild acidosis, as NaHCO_3_, an agent that can elevate pH, greatly compromised the neuroprotective effect of CO_2_. The authors found that CO_2_-induced acidosis inhibits mPTP and cytochrome c release, which can be abolished by the mPTP opener atractyloside, further demonstrating that CO_2_-induced Post-C is due to its acidic inhibition of mPTP.

### 6.6. Activation of the Mitochondrial ATP-Sensitive K^+^ Channel by Diazoxide

The mitochondrial ATP-sensitive K^+^ channel (mitoKATP) is a well-studied target for ischemic stroke tolerance and neuroprotection [96,97,98,99]. The neuroprotective effects of mitoATP in both Pre-C and Post-C have been evaluated. In Pre-C studies, diazoxide as the channel’s opener has been widely used [100]. Wu *et al*. [101] have reported that activation of mitoKATP by diazoxide 20 min before ischemia significantly improved neurological scores with a concurrent decrease in infarction volume. This protective effect could also be induced by cyclosporine A, which is an inhibitor of mPTP, demonstrating that mPTP closure following mitoKATP opening is involved in this protective response. Accordingly, the use of mPTP opener atractyloside diminished the neuroprotective effects of diazoxide and cyclosporine A.

In a Post-C study, Robin *et al.* [99] have reported that mitoKATP opening by diazoxide right before the start of reperfusion conferred significant neuroprotection. In this study, diazoxide was used in conjunction with ischemic Post-C comprising three episodes of 30 s of occlusion and reperfusion. The authors found that diazoxide resulted in a 60% decrease in infarction volume, and this effect was abolished by mitoKATP blocker 5-hydroxydecanoate (5-HD). Additionally, no delayed postconditioning effect was observed, as Post-C applied 5 min after the onset of reperfusion did not yield neuroprotection. However, in tissue culture studies, diazoxide was shown to trigger delayed Pre-C effects [98].

It should be noted that administration of diazoxide alone in the absence of post ischemic interruption of the reperfusion process has also been shown to confer neuroprotection [102]. Additionally, in addition to mPTP, ROS and calcium have been established as the mediators in diazoxide-induced neuroprotection [43].

### 6.7. Mitochondrial Biogenesis and Ischemic Tolerance

While numerous studies have focused on one protein target or one signaling pathway, mitochondrial biogenesis as a whole has also been investigated in the process of ischemic tolerance induced by chemical conditioning. For example, Stetler *et al*. [103] have reported that upon lipopolysaccharide (LPS)-induced preconditioning, mitochondrial biogenesis was observed, and this biogenesis is linked to ischemic tolerance. Many makers of mitochondrial biogenesis were found to be elevated by LPS, whose stroke tolerance effects have been well studied [104,105,106,107]. These markers include mitochondrial DNA copy number and mitochondrial transcription factor A (TFAM). The observation of mitochondrial biogenesis was further supported by TFAM knockdown, which attenuated mitochondrial biogenesis and ischemic tolerance induced by LPS preconditioning. This study demonstrates that mitochondrion, as an organelle, contributes to chemical-induced ischemic tolerance in the brain.

## 7. Summary and Future Perspectives

In this review, we have summarized evidence that chemical-induced ischemic stroke tolerance can be achieved by targeting mitochondrial proteins. We discussed a variety of targets, including complexes I, II, IV, ANT, mPTP and mitoKATP. Chemical agents that are covered in this review include CO, 3-NPA, CO_2_, isoflurane, diazoxide and cyanide. As some of these agents or their targets have not been tested in both Pre-C and Post-C settings, it would be interesting to evaluate their comprehensive effects on ischemic stroke tolerance in the future. Moreover, many mitochondrial proteins have not been explored as chemical conditioning targets for stroke tolerance, which should also be explored in the future. We believe that studies on elucidating the mechanisms of chemical-induced tolerance against stroke injury involving mitochondria as the target could eventually help fighting ischemic stroke, which is a leading cause of death globally.
